# Role of ^18^F-Fluorodeoxyglucose Positron Emission Tomography/Computed Tomography in the Diagnosis of Newly Found Suspected Malignant Solitary Pulmonary Lesions in Patients Who Have Received Curative Treatment for Colorectal Cancer

**DOI:** 10.1155/2017/3458739

**Published:** 2017-04-12

**Authors:** Xiaozhou Yu, Xiuyu Song, Lei Zhu, Wei Chen, Dong Dai, Xiaofeng Li, Wengui Xu

**Affiliations:** ^1^Department of Molecular Imaging and Nuclear Medicine, Tianjin Medical University Cancer Institute and Hospital, Tianjin, China; ^2^National Clinical Research Center for Cancer, Tianjin, China; ^3^Key Laboratory of Cancer Prevention and Therapy, Tianjin, China

## Abstract

*Background*. Positron emission tomography/computed tomography (PET/CT) is recommended for colorectal cancer (CRC) patients with suspected malignant pulmonary lesions. This study aims to systematically discuss the ^18^F-FDG-PET/CT diagnosis of solitary pulmonary lesions that are strongly suspected to be malignant in CRC patients who have previously undergone curative therapy. *Methods*. This retrospective study involved 49 consecutive CRC patients who had previously undergone curative therapy and then underwent PET/CT for the investigation of solitary pulmonary lesions that were strongly suspected to be malignant. *Results*. Pathological examination confirmed the presence of pulmonary metastases (29 patients, 59.2%), primary lung cancer (15 patients, 30.6%), and benign pulmonary disease (5 patients, 10.2%). Small lung lesions, advanced pathological stage, adjuvant chemotherapy after CRC surgery, solitary pulmonary lesions with lower border irregularity, higher carcinoembryonic antigen level, and the lack of concomitant mediastinal lymph node metastasis were more likely to be associated with pulmonary metastasis than with primary lung cancer. None of these factors was independently significant in the multivariate analysis. *Conclusion*. Clinicopathological characteristics help to differentiate metastasis and primary lung cancer to some extent during the diagnosis of solitary pulmonary lesions suspected to be malignant in this group of patients. This may provide valuable information to clinicians.

## 1. Introduction

Colorectal cancer (CRC) is a common malignancy with a high incidence of relapse. The relapse rate after standard treatments including surgery and chemotherapy has been estimated to be as high as 40% [1]. The lung is the second most common site of distant metastases from CRC [2, 3]. Thus, in CRC patients who have undergone curative treatment, the accurate diagnosis of suspected malignant lung lesions is crucial for treatment. ^18^Fluorine 2-fluoro-2-deoxy-d-glucose positron emission tomography and computed tomography (^18^F-FDG-PET/CT), a nuclear medicine technique, has been widely applied in the investigation of several malignancies [4–6]. The National Comprehensive Cancer Network (NCCN) guidelines for CRC therapy recommend ^18^F-FDG-PET/CT for the investigation of potentially curative metachronous pulmonary metastases [7–9].

Many authors have studied indeterminate lung lesions in CRC patients [10–12]. However, few have examined solitary pulmonary lesions in CRC patients. Furthermore, to our knowledge, no study has focused on the diagnosis of solitary pulmonary lesions by ^18^F-FDG-PET/CT in CRC patients who have previously undergone curative therapy. The diagnosis of solitary pulmonary lesions that are suspected to be malignant is challenging, because single pulmonary metastasis is rarer than multiple pulmonary metastases in CRC patients. Theoretically, a solitary pulmonary lesion could be attributable to metastasis, primary lung cancer, or benign disease. The diagnosis becomes challenging when the solitary pulmonary lesion shares some radiological features with primary lung cancer, such as border irregularity, lobulation, and spiculation. These overlapping features are frequently found in large solitary pulmonary lesions.

Solitary pulmonary lesions that are suspected to be malignant are a relatively rare but difficult-to-diagnose entity in CRC patients who have previously undergone curative treatment. In this retrospective study, we focused on ^18^F-FDG-PET/CT diagnosis. We aimed to identify relevant diagnostic features in these patients and to differentiate between solitary pulmonary lesions resulting from metastasis and those resulting from primary lung cancer.

## 2. Materials and Methods

### 2.1. Participants

**Figure 1:**
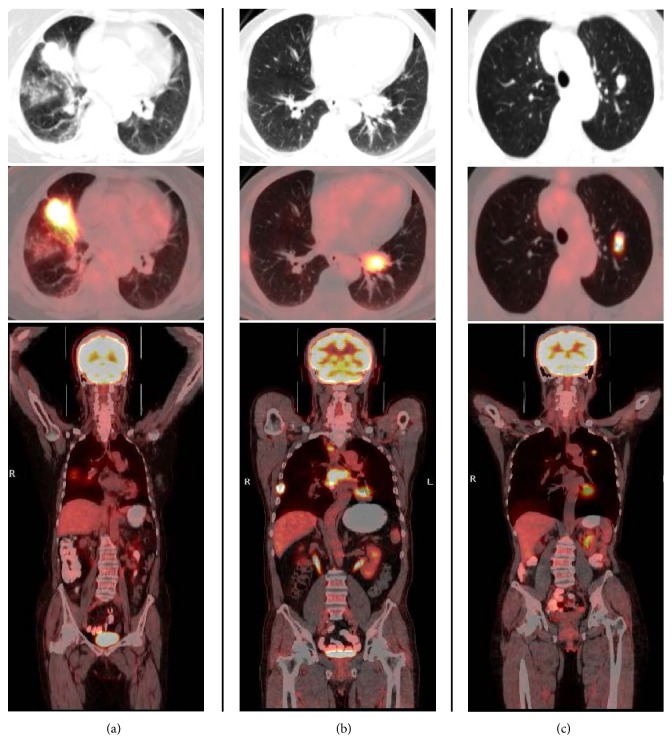
Representative ^18^F-FDG-PET/CT scans. (a) A 60-year-old woman had undergone surgery for rectal carcinoma (stage I) 103 months ago. ^18^F-FDG-PET/CT revealed a solitary pulmonary lesion that was suspected to be malignant (SUV_max_, 13.9). The CEA level was 3.7 *μ*g/L. The patient underwent pulmonary surgery. Pathological examination confirmed pulmonary metastasis. (b) A 65-year-old man had undergone surgery for colon carcinoma (stage II) 50 months ago, followed by adjuvant chemotherapy. PET/CT revealed a solitary pulmonary lesion that was suspected to be malignant (SUV_max_, 7.6), multiple mediastinal lymph metastases, and bone metastasis. The CEA level was 4.6 *μ*g/L. A bronchoscopic biopsy confirmed primary lung adenocarcinoma. (c) A 49-year-old woman had undergone surgery for colon carcinoma (stage II) 36 months ago, followed by adjuvant chemotherapy. PET/CT revealed a solitary pulmonary lesion that was suspected to be malignant (SUV_max_, 8.2). The CEA level was 5.3 *μ*g/L. Pulmonary surgery confirmed granulomatous disease. ^18^F-FDG, ^18^Fluorine-2-fluoro-2-deoxy-d-glucose; PET/CT, positron emission tomography/computed tomography; SUV_max_, maximum standardized uptake value; CEA, carcinoembryonic antigen.

This study was approved by the ethics committee of our center and is in accordance with the Declaration of Helsinki. Informed consent was waived, since this study was retrospective in nature. Forty-nine patients who underwent ^18^F-FDG-PET/CT scanning in our center between June 2009 and December 2015 were included. All participants had previously received curative treatment (surgery with or without adjuvant therapy), according to NCCN guidelines for CRC therapy. Patients underwent PET/CT scanning for the investigation of a newly found solitary pulmonary lesion that was highly suspected to be malignant on computed tomography (CT). Specifically, the solitary pulmonary lesion exhibited the following CT characteristics: large size (diameter, no less than 5 mm), with or without ill-defined borders, lobulation, and spiculation. None of the patients had any other pulmonary lesion or a history of lung metastasis. All patients were treated with either surgery or biopsy targeted to the lung lesion. Clinicopathologic data were collected and are shown in Table 1. Representative PET/CT scans are shown in Figure 1.

### 2.2. PET/CT Scanning and Image Interpretation

^18^F-FDG-PET/CT scanning was conducted using either a Discovery ST PET/CT scanner or a Discovery PET/CT 710 scanner (GE Healthcare, Milwaukee, WI, USA). Patients were required to fast for 6 h prior to the scanning. Before the intravenous injection of ^18^F-FDG, the glucose concentration of patients was measured. The administered activity of the radiotracer was 4.1–4.8 MBq (0.11–0.13 mCi) per kilogram of body weight. Scanning was performed from the midthigh to the vertex at approximately 1 h after ^18^F-FDG injection. CT scanning was performed with the following parameters: current, 120–170 mA; voltage, 120 kV; slice thickness, 5 mm or 3.75 mm; and reconstruction interval, 5 mm or 3.75 mm. Attenuation-corrected PET images were obtained at 2 min per bed and were reconstructed with iterative algorithms. The average number of beds was seven. In addition, a noncontrast CT scan targeted to the lung lesion with a slice thickness of 1.25 mm was also obtained from each patient. According to the degree of border irregularity, lobulation, and spiculation observed on noncontrast CT scans (slice thickness: 1.25 mm), lesions were visually scored from levels 1 to 5 as follows: level 1, lesions with regular borders; levels 2, 3, 4, and 5, lesions with slight, intermediate, slightly high, and high levels of lobulation and spiculation, respectively.

PET/CT images were viewed in the axial, coronal, and sagittal planes on a workstation (Xeleris, version 3.0562; GE Healthcare, Milwaukee, WI, USA). Regions of interest were manually drawn to define the standardized uptake values (SUVs). SUVs were generated automatically by the Xeleris software, according to the following equation: SUV = radioactivity concentration/(injected activity/bodyweight). Three senior nuclear medicine physicians with more than 10 years of experience independently interpreted these images and reached a diagnosis through consensus.

### 2.3. Carcinoembryonic Antigen Examination and Body Mass Index

The serum concentration of carcinoembryonic antigen (CEA) was measured in all patients by using the electrochemiluminescence method. The upper limit of normal was defined as 5.0 *μ*g/L. The body mass index (BMI) was calculated as the weight (kilograms) over the square of height (meters).

### 2.4. Statistical Analysis

Categorical variables were recorded as numbers and percentages. Continuous variables were recorded as median (range) or mean ± standard deviation (SD), depending on the distribution of data. Variable comparisons were performed using the independent-samples Student *t*-test, Mann-Whitney *U* test, or chi-square test. Logistic regression was used for multivariate analyses. All statistics were analyzed using SPSS 17.0 software (IBM, Armonk, NY, USA).

## 3. Results

### 3.1. Solitary Pulmonary Lesion and ^18^F-FDG-PET/CT

All patients had large, solid solitary pulmonary lesions (mean diameter: 2.3 ± 1.2 cm, range: 0.5–5.5 cm) that were strongly suspected to be malignant. The lesions were peripherally located in 45 (91.8%) patients and centrally located in 4 (8.2%) patients. On ^18^F-FDG-PET/CT, the lung lesion was ^18^F-FDG avid in 40 (81.6%) patients, with an SUV_max_ of >2.5. The mean SUV_max_ in all patients was 8.2 ± 5.4 (including solitary pulmonary lesions with both positive and negative SUVs). Of the nine patients with FDG-negative lesions, four patients had pulmonary metastasis, three had primary lung cancer, and two had benign diseases. In 12 (24.5%) patients, additional potentially malignant lesions were discovered on PET/CT. The following metastases were detected: mediastinal lymph node metastasis, 7 (14.3%) patients; local recurrence, 3 (6.1%) patients; pleural metastasis, 2 (4.1%) patients; bone metastasis, 5 (10.2%) patients; liver metastasis, 1 (2.0%) patient; supraclavicular metastasis, 2 (4.1%) patients; and cervical metastasis, 1 (2.0%) patient.

Among the 49 patients, 35 (71.4%) patients underwent pulmonary surgery and 14 (28.6%) underwent biopsy via either bronchoscopy or puncture. The results of the pathological examination of the samples were as follows: pulmonary metastasis, 29 (59.2%) patients; primary lung cancer, 15 (30.6%) patients; and benign pulmonary disease, 5 (10.2%) patients. Among the 15 patients with primary lung cancer, 9 (60%) had adenocarcinoma, 5 (33.3%) had squamous carcinoma, and 1 (6.7%) had large cell carcinoma. Furthermore, the 5 benign solitary pulmonary lesions were identified as sclerosing hemangioma, tuberculosis, inflammatory pseudotumor, granulomatous disease, and chronic inflammation.

### 3.2. Univariate and Multivariate Analyses

**Figure 2:**
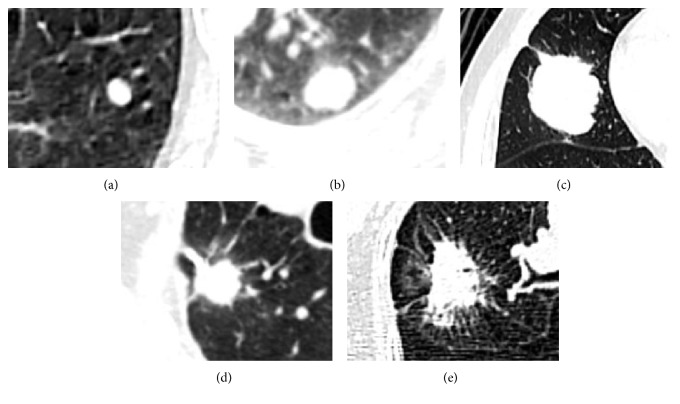
Solitary pulmonary lesions were visually classified into 5 levels according to the characteristics of their borders, as observed on noncontrast CT scans (slice thickness, 1.25 mm). The classification was performed by 3 senior physicians who reached a consensus for each patient. Representative lesions for levels 1–5 are shown. (a) A level 1 lesion with regular borders. (b) Level 2, (c) 3, (d) 4, and (e) 5 lesions with slight, median, slightly high, and high levels of lobulation and spiculation, respectively, and correspondingly ill-defined borders.

As shown in Tables 2 and 3, univariate analysis was conducted on factors that could potentially distinguish primary lung cancer from pulmonary metastasis. Small lung lesions, advanced CRC stage, adjuvant chemotherapy, lower border irregularity (Figure 2), higher CEA level, and lack of mediastinal lymph node metastasis were more likely to be associated with pulmonary metastasis than with primary lung cancer. Multivariate analysis indicated that none of the above factors was independently significant (Table 4).

## 4. Discussion

The NCCN guidelines for CRC therapy recommend the application of ^18^F-FDG-PET/CT in patients with potentially curative pulmonary metastases [7–9]. This investigation is also recommended for the diagnosis of suspected lung tumors [13, 14]. Thus, ^18^F-FDG-PET/CT is indicated in CRC patients who have undergone curative therapy and are then found to have solitary pulmonary lesions that are strongly suspected to be malignant. In CRC patients, the lung is the second most common site of distant metastases, with detection rates between 10% and 22% [11]. Studies have indicated that in CRC patients, almost all metachronous indeterminate pulmonary lesions are either metastases or benign pulmonary lesions, and their proportion varies dramatically depending on the selection criteria. For instance, Jung et al. found that 75% of CRC patients who had undergone surgical resection and then undergone lung surgery for suspicious indeterminate pulmonary lesions had metastases, and the remaining 25% had benign diseases [11]. However, an earlier study reported that benign conditions account for the majority of pulmonary lesions in this patient group [10].

CRC patients who undergo curative treatment and then develop a solitary pulmonary lesion that is highly suspected to be malignant are a relatively rare patient population, which has not been systematically studied before. On the one hand, pulmonary metastasis is quite common among CRC patients. On the other hand, these patients are also at a high risk of developing a second primary malignant disease, such as lung cancer [15, 16], which could be related to the genetic background, cancer treatment, lifestyle, and environmental risk factors [17]. Currently, due to a lack of evidence, the accurate diagnosis of solitary pulmonary lesions in this scenario is a great challenge for radiologists and clinical physicians. Although pathological results can be obtained from a considerable number of patients, an accurate radiological diagnosis or even a provisional preoperative diagnosis is critical and may help physicians formulate treatment plans. Furthermore, in patients who cannot undergo pathological examination, an evidence-based preoperative diagnosis is the only hope for proper treatment. In our patients, solitary pulmonary lesions were strongly suspected to be malignant due to their large size, with or without ill-defined borders, lobulation, and spiculation [18]. Thus, unsurprisingly, majority of the solitary pulmonary lesions (89.8%) in our study were confirmed to be malignant by pathological examination. However, intriguingly, approximately one-third of these malignant solitary pulmonary lesions were attributable to primary lung cancer, mainly primary lung adenocarcinoma, which is consistent with epidemiological reports [19]. In addition, approximately 10% of patients had benign lesions. Therefore, we strongly recommend the establishment of treatment strategies on the basis of pathological results, where possible.

Furthermore, we attempted to determine whether clinicopathologic data combined with ^18^F-FDG-PET/CT findings could help predict the results of the pathological examination of the lung lesions. This prediction would be critical in patients who are unable to undergo surgery or biopsy. We excluded benign solitary pulmonary lesions from this analysis, owing to their small sample size.

Univariate analysis indicated that small lung lesions, advanced pathological stage, adjuvant chemotherapy after CRC surgery, solitary pulmonary lesions with lower border irregularity, higher CEA level, and the lack of concomitant mediastinal lymph node metastasis were more likely to be associated with pulmonary metastasis than with primary lung cancer in our patient population. It is not surprising that advanced CRC stage was associated with metastasis, because pathological stage is significantly correlated with relapse and survival. The correlation between adjuvant chemotherapy and pulmonary metastasis may also be attributable to pathological stage. According to NCCN guidelines, chemotherapy is not recommended for patients with stage I CRC and some patients with stage II CRC. In contrast, almost all patients with stage III and IV CRCs are recommended adjuvant chemotherapy if there are no contraindications to this treatment.

We also found that the diameter of primary lung cancers was significantly higher than that of pulmonary metastasis in our patient population. In addition, primary lung cancers tended to have more irregular borders than pulmonary metastases, although in this study, most solitary pulmonary lesions had ill-defined borders with varying degrees of lobulation or spiculation.

CEA level was also a significant factor in the univariate analysis. The majority of CRC patients have elevated serum CEA levels [20]. CEA has been highly recommended as a marker of CRC in multiple clinical guidelines and is widely used in the surveillance of CRC patients after curative treatment [8, 9, 20]. However, some lung cancer patients also have increased serum CEA levels; and CEA is used as a tumor marker in lung cancer management, although its sensitivity is relatively low [21, 22]. Considering the more dominant role of CEA in CRC rather than in lung cancer, we consider that this marker may be a useful diagnostic tool.

Metastasis patterns differ between CRC and lung cancer. In lung cancer patients, local metastasis tends to involve the mediastinal lymph nodes, whereas distant metastases can involve organs such as the bone, adrenal glands, liver, and brain. In CRC patients who have undergone curative treatment, common sites of metastasis are the local lymph nodes, liver, and lungs. ^18^F-FDG-PET/CT has been shown to have high sensitivity and specificity for the detection of concomitant metastasis in patients with CRC or primary lung cancer [13, 14, 23, 24]. Therefore, we evaluated potential concomitant metastases in our patients by using ^18^F-FDG-PET/CT. According to our data, patients with concomitant mediastinal lymph node metastases are more likely to be diagnosed with primary lung cancer than with CRC metastases. However, we did not find sufficient evidence to prove the diagnostic role of this technique in other concomitant metastases.

In addition, we also analyzed a series of potential diagnostic and clinicopathologic factors. However, none of these factors was a significant independent predictor of metastasis. Some studies have indicated that CRC is related to obesity and that BMI could be a risk factor for its occurrence, progression, and metastasis and for patient survival [25–27]. In contrast, overweight has been reported to play a protective role in lung cancer [28]. Our study revealed that BMI was not a significant factor to distinguish primary lung cancer from pulmonary metastasis in our patient population.

It has also been reported that the pulmonary metastasis of CRC is more commonly seen in patients with rectal cancer than in patients with colon cancer [11]. A possible reason is the closer connection of the rectal veins, rather than the portal vein, to the systemic circulation. However, this factor was not diagnostic in our study.

Time since surgery has also been suspected to be a predictive factor to distinguish metastasis and primary lung cancer, because metastasis should roughly follow a Kaplan-Meier curve. However, this factor was not significant in our study.

As expected, the location of the solitary pulmonary lesion (central versus peripheral) was also not diagnostic. This is because although most pulmonary metastases in CRC patients are located in the periphery of the lung [11], the majority of primary lung cancers also occur in peripheral sites.

Both primary lung cancer and CRC metastasis are highly malignant tumors. Thus, they tend to have high glycometabolism. SUV is one of most widely used semiquantitative indicators in ^18^F-FDG-PET/CT analysis. As expected, there was no significant difference in the rate of positive FDG uptake and the level of FDG absorption between primary lung cancer and CRC metastasis.

In our study, only 5 patients were found to have tumor recurrence after CRC surgery, and none of these recurrent tumors involved the lungs. All 5 patients received additional treatment for the recurrent tumors and subsequently presented with solitary pulmonary lesions. There is no doubt that CRC patients who present with a solitary pulmonary lesion after developing tumor recurrence have a poorer prognosis than patients who do not develop tumor recurrence. Thus, although the evidence is not currently sufficient, we suspect that CRC patients with recurrence are more likely to have pulmonary metastasis than primary lung cancer.

Since none of the above factors was an independent significant diagnostic factor on the multivariate analysis, these factors must be comprehensively analyzed in patients who cannot undergo surgery or biopsy, in order to estimate the pathological result.

In conclusion, most solitary pulmonary lesions that develop after curative treatment in CRC patients are pulmonary metastases, followed by primary lung cancer and benign pulmonary disease. Wherever possible, treatment strategies should be based on the outcomes of the pathological examination of the solitary pulmonary lesion. Although PET/CT was of limited use in differentiating metastasis from primary lung cancer, considering its other more valuable uses, such as in cancer staging, it is very likely to be performed in these patients according to current practice guidelines. Certain clinicopathological characteristics, to some extent, aid in the differentiation of CRC metastasis and primary lung cancer. As a whole-body scan with high accuracy in both CRC and lung cancer, PET/CT can detect the tumor distribution pattern. However, in this study, only mediastinal lymph node metastasis could aid in preoperative diagnosis; tumors in other locations, such as skeletal metastasis, local recurrence, and liver metastasis, had a limited role in the diagnosis of solitary pulmonary lesions in our patient population. Advanced pathological stage, adjuvant chemotherapy after CRC surgery, solitary pulmonary lesions with lower border irregularity, higher CEA level, and the lack of concomitant mediastinal lymph node metastasis are more likely to be associated with pulmonary metastasis than with primary lung cancer. These factors may be considered in patients who are not fit enough to undergo surgery or biopsy and may provide valuable information to clinical physicians.

## Figures and Tables

**Table 1:** Patient characteristics.

Variable	*N*	%
Gender		
Male	31	63.3
Female	18	37.7
Administered activity of tracer, MBq (mCi), median (range)	311 (196–426), 8.4 (5.5–11.7)	
Age, years, median (range)	62 (43–77)	
Primary tumor site		
Colon	21	42.9
Rectum	28	57.1
T stage		
T1	1	2.0
T2	5	10.2
T3	6	12.2
T4	37	75.5
N stage		
N0	35	71.4
N1	10	20.4
N2	4	8.2
Pathological stage		
I	6	12.2
II	28	57.1
III	11	22.4
IV^∗^	4	8.2
Treatment		
Adjuvant chemotherapy		
Yes	38	77.6
No	11	22.4
Adjuvant radiotherapy		
Yes	7	14.3
No	42	85.7
Time since surgery, months, median (range)	30 (3–235)	

^∗^All 4 patients had stage IVA disease with liver metastasis and had received curative treatment.

**Table 2:** Characteristics of patients with lung metastases and primary lung cancer.

Variable	Primary lung cancer	Lung metastasis	*P* value
Sex			0.146
Male	12	17	
Female	3	12	
Age, years, median (range)	65 (49–77)	62 (48–77)	0.211
BMI, median (range)	25.7 (20.3–30.8)	24.5 (18.2–36.3)	0.833
Primary tumor site			0.459
Colon	5	13	
Rectum	10	16	
Pathological stage			0.003
I-II	14	15	
III-IV	1	14	
Treatment			
Adjuvant chemotherapy			0.019
Yes	8	26	
No	7	3	
Recurrence			0.149
Yes	0	5	
No	15	24	
Time since surgery, months, median (range)	23 (4–144)	30 (3–104)	0.901

BMI: body mass index.

**Table 3:** Characteristics of lung lesions in patients with lung metastasis and primary lung cancer.

Variable	Primary lung cancer	Lung metastasis	*P* value
Size of lung lesion, cm	2.8 ± 1.2	2.1 ± 1.1	0.045
Morphological score of lung lesion	3.5 ± 0.8	2.7 ± 1.0	0.010
SUV_max_ of lung lesion, median (range)	12.1 (1.1–23.5)	7.2 (0.6–19.4)	0.207
FDG uptake			0.675
Positive	12	25	
Negative	3	4	
Position			0.481
Peripheral	13	27	
Central	2	2	
Metastasis detected on PET/CT			
Mediastinal lymph node metastasis			0.004
Yes	6	1	
No	9	28	
Skeletal metastasis			0.319
Yes	3	2	
No	12	27	
Local recurrence			1.000
Yes	1	2	
No	14	27	
Liver metastasis			1.000
Yes	0	1	
No	15	28	
CEA concentration, μg/L, median (range)	2.4 (1.0–73.3)	4.8 (1.7–69.7)	0.005

SUV: standardized uptake value; FDG: ^18^Fluorine-2-fluoro-2-deoxy-d-glucose; PET/CT: positron emission tomography/computed tomography; CEA: carcinoembryonic antigen.

**Table 4:** Multivariate analysis.

Variable	OR (95% CI)	*P* value
Pathological stage	0.104 (0.009–2.127)	0.157
I-II		
III-IV		
Adjuvant chemotherapy	5.624 (0.779–40.595)	0.087
Yes		
No		
Morphological score of lung lesion	0.660 (0.249–1.745)	0.402
Size of lung lesion, cm	0.736 (0.357–1.514)	0.404
Mediastinal lymph node metastasis	47.978 (0.950–2422.772)	0.053
Yes		
No		
CEA concentration, ng/L	1.031 (0.957–1.112)	0.422

OR: odds ratio; CEA: carcinoembryonic antigen; CI: confidence interval.
